# Neuroinflammation in Dementia—Therapeutic Directions in a COVID-19 Pandemic Setting

**DOI:** 10.3390/cells11192959

**Published:** 2022-09-22

**Authors:** Mateusz Łuc, Marta Woźniak, Joanna Rymaszewska

**Affiliations:** 1 Department of Psychiatry, Wroclaw Medical University, 50-367 Wroclaw, Poland; 2 Department of Pathology, Wroclaw Medical University, 50-367 Wroclaw, Poland

**Keywords:** neuroinflammation, TNF, SARS-CoV-2, COVID-19, glial cells, TNF antagonists

## Abstract

Although dementia is a heterogenous group of diseases, inflammation has been shown to play a central role in all of them and provides a common link in their pathology. This review aims to highlight the importance of immune response in the most common types of dementia. We describe molecular aspects of pro-inflammatory signaling and sources of inflammatory activation in the human organism, including a novel infectious agent, SARS-CoV-2. The role of glial cells in neuroinflammation, as well as potential therapeutic approaches, are then discussed. Peripheral immune response and increased cytokine production, including an early surge in TNF and IL-1β concentrations activate glia, leading to aggravation of neuroinflammation and dysfunction of neurons during COVID-19. Lifestyle factors, such as diet, have a large impact on future cognitive outcomes and should be included as a crucial intervention in dementia prevention. While the use of NSAIDs is not recommended due to inconclusive results on their efficacy and risk of side effects, the studies focused on the use of TNF antagonists as the more specific target in neuroinflammation are still very limited. It is still unknown, to what degree neuroinflammation resulting from COVID-19 may affect neurodegenerative process and cognitive functioning in the long term with ongoing reports of chronic post-COVID complications.

## 1. Introduction

Although dementia is a heterogenous group of diseases, inflammation has been shown to play a central role in all of them and provides a common link in their pathology. This review aims to highlight the importance of immune response in the most common types of dementia, discuss essential mechanisms and sources of neuroinflammation and potential therapeutic strategies.

Prolonged activation of pro-inflammatory responses in Alzheimer’s disease (AD) alters function of glial cells and in turn, further accelerates neuroinflammation [1]. Subsequent synaptic dysfunction and loss of neurons are responsible for clinical symptoms of the disease. Additionally, factors such as insufficient sleep length and subsequent reduction in amyloid clearance via the glymphatic system lead to amyloid accumulation, while simultaneously aggravating systemic inflammatory response [2]. Inflammation in vascular dementia (VaD) contributes to the three-hit hypothesis, along with hypertension and hypoxia [3]. Vasculitis is responsible for restricted blood circulation in microvessels and leads to decreased oxygen supply and regional glial activation favoring neuroinflammation in the central nervous system (CNS) [4,5]. In the following cascade, blood vessels undergo remodeling, the blood-brain barrier (BBB) becomes more permeable and microthrombi cause regional hypoxia and neural death [3,4,6]. While neuroinflammation in frontotemporal dementia (FTD) is evident with both pro- and anti-inflammatory cytokines levels increased in the brain, the impact of the systemic inflammatory activation remains inconclusive [7]. However, some studies indicate that FTD is associated with autoimmune activation and more frequent comorbidities such as thyroid and rheumatoid diseases but also with elevated TNF concentrations [8,9] These observations reflect the need to account for peripheral immune response in understanding pathology of the disease [10]. Lewy bodies, α-synuclein aggregates present in neurons are a hallmark trait of Lewy body dementia (LBD) [11]. Microglial phagocytosis of α-synuclein aggregates leads to a release of pro-inflammatory cytokines, such as IL-6, contributing to increased iron sequestration in neurons and exacerbating neural death [12]. α-synuclein acts as an agonist of microglial Toll-like Receptor 2 (TLR-2) resulting in oxidative stress and production of TNF, IL-1 and IL-6 [13]. Addressing evidence from Parkinson’s disease studies, extracellular α-synuclein stimulates leucine rich repeat kinase 2 (LRRK2) expression in monocytes, favoring their infiltration of the brain parenchyma [14,15]. Additionally, α-synuclein oligomers induce release of calcium from astrocytes leading in turn to glutamatergic neurotoxicity and synapse loss [16].

As briefly discussed above, source and role of inflammation varies widely depending on specific dementia type. However, recent research highlights the role of peripheral cytokines and immune cells in the neurodegenerative processes [17,18,19]. Numerous comorbidities, which are considered to be risk factors of cognitive decline, are linked to dementia pathology via several mechanisms, among which, inflammation plays a significant role. While some studies detected a small association between inflammatory markers and global cognition in the elderly or function disability in dementia patients, life-long immune response could not be accounted for in any of those studies [20,21,22]. Hence, it is currently argued that the total impact of systemic life-long pro-inflammatory activation, such as occurs in obesity or rheumatoid diseases, and its role in cognitive decline require further exploration. In the following sections we describe molecular aspects of pro-inflammatory signaling and sources of inflammatory activation in the human organism, with a special regard to a novel infectious agent, SARS-CoV-2 (severe acute respiratory syndrome coronavirus 2). The role of glial cells in neuroinflammation, as well as potential therapeutic approaches, are discussed.

## 2. Tumor Necrosis Factor Triggers Dementia Pathology

Among various cytokines involved in the immune response, tumor necrosis factor (TNF) is considered to play a significant upstream role in dementia pathology on a molecular level. Its pleiotropic effects vary from physiological neuroprotective and repair activities to pathological neuronal loss occurring in neurodegenerative and autoimmune conditions which are dependent on TNF form and activated receptor [23]. TNF receptor type 1 (TNFR1) is commonly expressed and can be bound by both transmembrane and soluble TNF forms, while TNF receptor type 2 (TNFR2), expressed by myeloid and endothelial cells but also by CNS-residing glia and neurons, is mainly bound by transmembrane TNF [24]. Protective versus deleterious outcomes of receptor activation depend on various factors including TNF concentration, activation of other signaling pathways or cell susceptibility resulting from cell type and age-related priming [25,26]. TNF signaling activates intracellular pathways with transcription factors such as NFκB (nuclear factor kappa-light-chain-enhancer of activated B cells) leading to pro-inflammatory cytokine production and, in conditions of prolonged signaling, aggravation of inflammation [27,28].

Exposure to TNF in an in vitro AD model has been shown to result in aggregation of extracellular proteins which are considered characteristic traits of AD and LBD pathology [29]. Interestingly, exposure to TNF does not need to be sustained in order to maintain increased secretion and aggregation of amyloid-β or α-synuclein in such models. This mechanism may demonstrate profound effects of TNF signaling on pro-inflammatory activation and cytokine production of astrocytes and microglia, leading to prolonged neuroinflammation [30]. This phenomenon has been reflected in a cohort study by Lindbergh et al., in which TNF plasma concentration of participants were assessed annually. Increased systemic TNF resulted in reduction in grey matter volumes in further assessments in a curvilinear correlation, with initially-increased TNF correlating with following loss of volume [31]. Additionally, within-person increases in TNF correlated with lower scores obtained in neuropsychological evaluation with the use of Mini Mental State Examination.

## 3. Mechanisms of Chronic Low-Grade Inflammation

Chronic low-grade inflammation stems from various conditions such as obesity, autoimmune and metabolic diseases, but also from psychosocial stressors. Adipokines released by white adipose tissue favor low-grade chronic inflammation and it has been shown that resulting changes to cytokine levels in mouse hippocampi can be elicited by simple fat tissue transplantation [32,33]. Obesity in humans is associated with greater neuroinflammation and worse cognitive performance, as shown by Samara et al., in a cohort study [34]. The impact of obesity on cognition is mediated both by adipokine dysfunction and increased production of pro-inflammatory cytokines by activated adipocytes [35,36]. Rheumatoid diseases are linked to a higher risk of atherosclerotic lesions and the proposed mediator is low-grade inflammation [37]. Both metabolic syndrome and type 2 diabetes have also been implicated in low-grade inflammation [38]. Moreover, in a longitudinal study chronic work-related stressors were associated with increased inflammatory index in men [39].

Another important factor in pro-inflammatory activation of the immune system seems to be composition of gut microbiota, which interestingly, has also been associated with above-mentioned causes of chronic inflammation [40]. Recent studies highlight the influence of diverse and complex environments present in the gastrointestinal tract on various health outcomes, including neurodegenerative diseases [41]. The bidirectional relations between gut microbiota and brain are reflected by the term gut-brain axis in which both blood and vagus nerve serve as mediators. In Parkinson’s disease α-synuclein aggregates are detected in intestinal submucosal plexus in a prodromal stage of disease [42]. Additionally, pathological α-synuclein was shown to be reversely transported via vagus nerve in animal models [43]. A similar mechanism has also been hypothesized for amyloid-β in AD [44]. The noteworthy mechanisms of microbial impact on the CNS include (1) intestinal production of cytokines; (2) entry of bacterial toxins, such as lipopolysaccharides, to the bloodstream; (3) microbial production of neurotransmitters; (4) direct passage of microbes to bloodstream and reactive production of cytokines by immune cells, but also (5) microbial entry to the CNS. Several highly adapted bacterial species, such as *Streptococcus pneumoniae* or *Neisseria meningitidis* are able to cross the BBB, often leading to its disruption and clinical manifestations of neurological infection. However, most of the microbial interactions with the CNS are considered to occur in a chronic, life-long fashion [45,46]. On the other hand, it is argued that activation of hypothalamic-pituitary-adrenal axis by circulating lipopolysaccharides constitutes CNS response and affects the gastrointestinal tract in a feedback loop manner [47].

## 4. SARS-CoV-2—A Novel Source of Neuroinflammation?

Recently, global exposure to the novel infectious agent, SARS-CoV-2, raises a question about neuroinflammatory consequences of COVID-19 (Coronavirus Disease 2019). Despite the fact that most cases of the infections are mild, their long-term effects in humans remain unknown [48]. SARS-CoV-2 has proven neurotropism and was shown to enter the CNS and disrupt the BBB integrity [49,50,51,52]. The entry of the virus to the CNS could occur via the olfactory nerve retrograde route but also with viral particles infecting endothelial cells and in conditions of BBB impairment, also pericytes and astrocytes [53,54].

So far, insufficient data exists on hypothesized retrograde axonal transport of the SARS-CoV-2. However, potential cellular mechanisms for this pathway could include ESCPE-1 retrograde trafficking. This endosomal transport system is responsible for sorting of neuropilin-1, which was shown to be a host factor for SARS-CoV-2 infection but further studies are required in order to confirm this pathway [55]. While evidence exists that virus present in the blood infects endothelial cells causing loss of the BBB integrity and allowing for entry to the CNS [56,57], it is still unclear whether disruption of BBB occurs due to tight junctions alterations [58] or basement membrane remodeling [59]. Another hypothesized pathway is the ‘Trojan horse’ mechanism observed in HIV infection, with infected circulating macrophages crossing the BBB and transporting SARS-CoV-2 to the brain compartment [60].

The apolipoprotein E4 (ApoE4) genotype has been associated with decreased antiviral defense gene expression resulting in increased risk of neuronal or astrocytic infection and more aggravated inflammatory response in astrocytes [61,62]. This finding links COVID-19 to AD pathology, for which ApoE4 gene is a well-established risk factor. Brain-residing cells, such as neurons, glia or pericytes have been shown to express the angiotensin-converting enzyme (ACE2) receptor, which facilitates viral infection in other organs such as lungs or heart [63,64,65]. Studies conducted in vitro indicate that SARS-CoV-2 infection in neurons results in synapse loss along with a decreased number and impaired morphology of neurites [61]. Furthermore, a multimodal omics approach revealed correlations between COVID-19 neuroinflammation and cognitive decline, with special emphasis on AD microvascular injury pathways [62].

**Figure 1:**
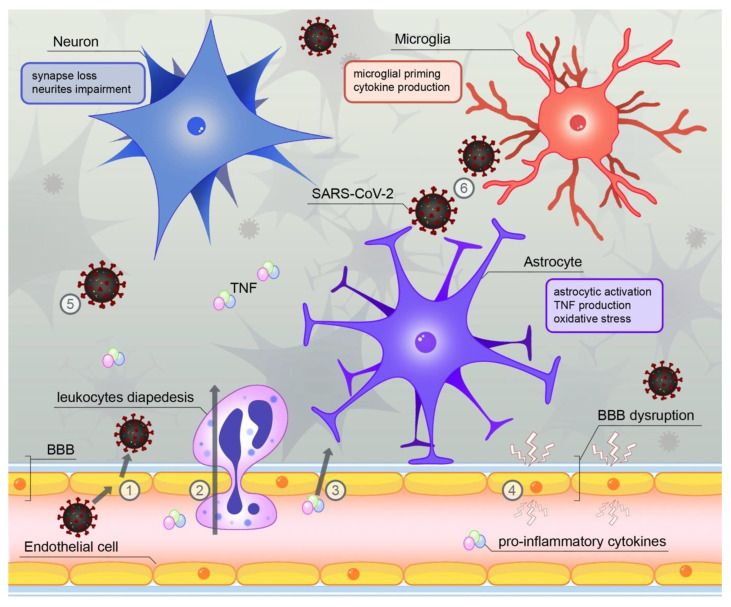
SARS-CoV-2 infection impacts the CNS via several potential pathways: (1) Infection of endothelial cells by viral particles present in the blood; (2) infiltration of the CNS by activated leukocytes from the bloodstream; (3) entrance of pro-inflammatory cytokines into the CNS; (4) loss of the BBB integrity due to increased immune response; (5) hypothetic direct passage of viral particles via olfactory nerve and (6) activation of pro-inflammatory phenotypes of CNS-residing cells.

While acute infection has been documented to cause neurological conditions such as encephalopathy and meningoencephalitis, their occurrence does not require the viral invasion of the CNS but can also result from other neuroinflammatory pathways [66]. In a study of 29 COVID-19 patients with neurological manifestations of the disease, most of them did not test positively for SARS-CoV-2 RNA in the cerebrospinal fluid (CSF) samples [67]. The total impact of the inflammatory activation in the CNS by SARS-CoV-2 is yet to be described. Peripheral immune response and increased cytokine production, including an early surge in TNF and IL-1β concentrations, impact the CNS-residing cells and favor their priming [53,54,66,68,69,70,71] (Figure 1). One of the proposed mechanisms of this phenomenon is entry of the peripheral pro-inflammatory signaling to the CNS via endothelial cells [72]. The effects of systemic inflammation observed in the brain compartment during COVID-19 have been described in several reports in which CSF pro-inflammatory cytokines levels were elevated throughout the course of the disease [73,74]. Additionally, cardiovascular consequences of COVID-19, such as coagulopathy and stroke, also contribute to cognitive decline [68,70,75]. As it turns out, despite some reports of viral presence in the CNS, the relevant consequences of COVID-19 in regard to neurodegeneration may actually occur without infiltration or replication of SARS-CoV-2 in the brain compartment. So far, a novel somatic symptomatology similar to chronic fatigue syndrome has been reported and described as post-COVID syndrome or long COVID [76], but other long-term consequences of COVID-19 may be reported in the future and more mechanistic studies are required in order to answer which pathways are of importance in their development.

## 5. Glial Involvement in Neuroinflammation

Research shows that the physiological neuroprotective immune response becomes impaired in conditions of prolonged pro-inflammatory activation in the CNS. The intricate interplay between different cell types residing in the CNS, such as neurons, microglia or astrocytes, becomes disturbed, eventually altering their function and leading to progressive aggravation of molecular pathology. Glial cells take part in nourishment of neurons but also affect their maturation, synapse formation and proper function [77,78]. It has been shown that both astrocytes and microglia exhibit neuroprotective and neuroinflammatory phenotypes, though these two main phenotypes may vary widely [79,80,81]. Loss of neuroprotective glial functions may in fact be associated with age-related decrease in acetylcholine receptors which are responsible for anti-inflammatory glial actions [80,82].

Astrocytes, the most numerous cells in the CNS maintain proper neurotransmission, regulate neural metabolism and oxidative status and contribute to glymphatic clearance of deleterious substrates [83,84]. Along with neurons and endothelial cells they allow for neurovascular coupling crucial for separation but also equilibrium maintenance in blood and brain compartments [85]. However, in neurodegenerative diseases their neuroprotective and neuroregulatory roles are impaired. Evidence indicates that astrocytes exhibit more pro-inflammatory phenotype with older age [86]. Exposure to phosphorylated tau oligomers evokes a pro-inflammatory astrocyte phenotype in AD and FTD patients resulting in a further increase in TNF production and activation of inflammatory phenotypes in surrounding cells [87]. Additionally, activated astrocytes are implicated in amyloid production increasing amyloid burden in the CNS, while their effectiveness in amyloid clearance decreases with time [79]. In LBD, α-synuclein aggregates accumulate in astrocytes leading to their chronic activation [83]. Microangiopathy impairs function of the neurovascular unit. In such conditions, astrocytes lose their buffering function leading to potassium imbalance and altered neuronal excitability [88]. In an animal model of VaD, reactive astrocytes significantly influenced survival of hypoxic neurons, which was mediated by lipocalin-2 expression [89]. Use of human-induced pluripotent stem cells and their differentiation into astrocytes led to remyelination and axonal sprouting enabling improvement of cognitive functions in another rodent model of VaD [90].

Microglia are primary immune cells responsible for phagocytosis, cytokine production and immune surveillance in the CNS [91,92]. However, their longevity and low turnover facilitate development of age-related neurodegenerative diseases [93]. It is currently believed that throughout life microglia respond to various stressors and become primed leading to often exaggerated and prolonged inflammatory responses to stimuli present in older age [94,95,96]. Primed microglia are characterized by less efficient amyloid phagocytosis and greater production of pro-inflammatory cytokines, such as TNF [97,98]. Interestingly, TNF has been shown to inhibit microglial clearance and increase production of amyloid in the CNS [99]. The primed microglia are also characterized with overexpression of immune surface protein TREM 2 (triggering receptor expressed on myeloid cells 2), which initially helps to alleviate amyloid burden [80,100]. In the brain compartment TREM2 is expressed by microglia only and promotes their survival, activation and phagocytosis [101,102]. CSF concentration of its soluble form, sTREM2, is indicative of neurodegeneration, with its higher levels correlating with slower cognitive decline in AD patients [103]. TREM gene variants have been implicated in pathologies of AD, FTD, along with α-synucleinopathies [101]. However, conflicting results from animal studies lead to the conclusion that the role of TREM2 in dementia pathology may actually be dependent on the stage of disease, with TREM2 reducing amyloidogenesis at early stages but eventually increasing development of amyloid plaques [104,105,106,107]. The reasons for the changed outcome of this signaling pathways may result from prolonged peripheral inflammatory activation influencing the CNS and therefore, activation of other signaling pathways.

Apparently, impaired microglial function resulting in ineffective amyloid plaque clearance favors development of microgliosis. In neurodegenerative diseases such as AD and LBD, the total number of microglia is increased, while number of activated microglia correlates with observed tau pathology [99,108]. Tau aggregates, have in turn been shown to induce NLRP3 inflammasome activation, leading to further production of IL-1β by microglia and aggravation of neuroinflammation [109]. Of note, pharmacological reduction in microgliosis and alteration of the glial pro-inflammatory phenotype leads to alleviation of tau-related pathology and improvement of cognitive functioning in an animal model [110]. Evidence exists, that activated glia may in fact facilitate propagation of α-synuclein pathology in in vitro models of α-synucleinopathies [111,112,113]. Additionally, microglia and astrocytes have been implicated in defective autophagy and glutamate excitotoxicity in FTD [114,115,116].

## 6. Methods for Reduction of Pro-Inflammatory Activation—Critical Appraisal

Since TNF signaling exerts a triggering effect in the development of cognitive decline [29,31], the question arises, whether interventions focused on reduction in TNF concentrations or inhibition of its signaling pathway may play a role in dementia prevention or treatment. There are several methods of reducing an inflammatory state in the human organism, including dietary and pharmacological interventions.

Diet has been implicated in pathology of numerous neurodegenerative diseases, including dementia. A well-balanced diet provides all nutritional ingredients necessary for maintaining healthy and functional neurons [117]. On the other hand, it is believed that diet largely impacts inflammatory activation and immune response, which in turn influences regional inflammatory response in the brain. Various studies, including large cohorts, have associated inflammatory dietary patterns with faster cognitive decline and subsequent cognitive impairment [118,119,120]. The correlation between inflammatory diet and cognition is especially apparent in regard to several cognitive functions, such as episodic memory, semantic-based memory, executive functions and working memory [121].

The Western diet which comprises highly-processed food rich in fructose and saturated fat is known to increase TNF concentration in animal models [122]. Mice immunized against *Klebsiella pneumoniae* were shown to have lower levels of inflammasome-related inflammation, providing yet another link between gut microbiota and inflammation. This phenomenon was mediated by the presence of apolipoprotein E and was not observed in ApoE −/− animals [122]. Although individually tailored diets are not yet within reach and require further exploration, some conclusions regarding the influence of particular diets on neurodegenerative processes can be made based on existing studies [123]. For example, use of Dietary Inflammatory index (DII), which takes into account individual’s dietary composition and characteristics, allows us to indicate the general influence of one’s diet on systemic cytokine levels and inflammatory activation [124]. Higher scores of DII were shown to strongly correlate with worse cognitive performance [125]. It stands to reason, that the influence of environmental factors such as diet in preserving cognition cannot be underestimated. On the other hand, it seems that the inflammatory potential of the consumed food may be related not only to the specific products but also to the gut microbiota composition resulting from the daily diet. A summary of findings associated with specific diets can be found in Table 1 [126,127,128,129,130,131,132,133,134,135].

Non-steroidal anti-inflammatory drugs (NSAIDs), the most commonly used anti-inflammatory drugs, were considered as potential dementia preventive agents in numerous studies. Some animal and human cohort studies suggested a beneficial influence of a daily intake of specific NSAIDs in preventing development or progression of most common types of dementia via reducing distinct dementia pathology [136,137,138]. However, large randomized clinical-trials or meta-analyses did not provide strong evidence supporting their recommended use, with a special regard to the dangerous side effects of their daily intake, such as potential gastrointestinal bleeding [139,140,141,142,143,144,145,146].

TNF antagonists offer yet another interesting approach to reducing inflammatory activation in humans. These agents are commonly used in autoimmune diseases, such as rheumatoid arthritis, psoriasis or inflammatory bowel diseases [147]. Their effects in controlling excessive immune response are mediated by binding TNF but the exact mechanisms and affinity to soluble and transmembrane TNF vary, hence their clinical use can also differ [26,148,149]. Moreover, some other mechanisms of action have been described, such as lymphotoxin-α blocking by etanercept. Additionally, infliximab has been proven to reduce expression of GM-CSF (granulocyte-macrophage colony stimulating factor) [150,151], while infliximab and adalimumab are able to induce production of immunosuppressive IL-10 by macrophages in vitro [152]. Psoriasis patients treated with etanercept had decreased expression of IL-1 and IL-8 genes which correlated with reduction in total pro-inflammatory immune response [153]. Importantly, although molecular weight and properties do not allow for free entry of TNF antagonists into the CNS, evidence indicates that these drugs may have protective influence against brain aging.

It seems that both BBB-nonpenetrating and modified, BBB-penetrating, etanercept reduce tauopathy, microgliosis and therefore, neuronal loss in a mouse model of AD. Additionally, they increase PSD95 protein levels indicating synaptic health. This phenomenon may be related to the peripheral effects of the drug and underlines the importance of tackling chronic inflammatory activation in order to maintain physiological neuronal function [154]. Similar observations had been made previously, in a study by Chang et al. [155]. Administration of BBB-penetrating TNF-inhibitor, cTfRMAb-TNFR, resulted in a significant decrease in neuroinflammatory markers, amyloid burden and BBB disruption in an AD mouse model. These results were comparable to those obtained with the use of etanercept in regard to amyloid burden and BBB integrity but not for neuroinflammation portrayed with ICAM-1 concentration. The cognitive performance of tested mice was highest in a group treated with cTfRMAb-TNFR highlighting the crucial role of neuroinflammation in cognitive decline [155]. Use of BBB-penetrating agents remains especially interesting in regard to FTD pathology, in which cytokine production seems to take place mainly in the brain [7].

An experiment conducted in an animal model of metabolic syndrome proved that intraperitoneal administration of infliximab improved lipid profiles in rats—i.e., decreased triglycerides and increased HDL. Additionally, the study group had lower adiponectin concentrations compared to the control, impacting low-grade inflammation. Noteworthy, not all metabolic aspects were normalized and cognitive tests were not included in the study protocol [156]. It may be hypothesized that an up-stream role of TNF in obesity-induced pathology, limits the potential protective effects of TNF inhibition in conditions of already present metabolic syndrome. On the other hand, intracerebroventricular administration of infliximab in a transgenic mouse model of AD resulted in a significant decrease in brain TNF levels, reduction in amyloid burden and tau pathology [157]. Another study conducted in a rat model of VaD, revealed a therapeutic effect of adalimumab administration in treating cognitive deficits resulting from cerebral hypoperfusion. This finding was associated with a reduction in neuronal loss and of microglial activation mediated by NFκB suppression [158].

A systematic literature review of TNF antagonist effects on AD revealed beneficial influence of TNF inhibition on cognition [27]. Most commonly studied agents, etanercept, infliximab and adalimumab, used in rheumatoid arthritis coincided with up to 60–70% reduction in AD incidence in large epidemiological analyzes of rheumatoid arthritis patients, whereas other methods of treatment did not affect AD incidence [159]. Similar results were obtained in patients with psoriasis who were treated with etanercept, infliximab or adalimumab [160]. Rheumatoid arthritis and psoriasis, among other autoimmune diseases, are known risk factors of AD due to the occurrence of persistent inflammatory activation which may constitute a confounding factor for generalized conclusions [161,162,163]. However, these observations warrant further research in other populations in order to establish a protective role of TNF inhibition on development and progression of various types of dementia. Updates on current clinical trials can be found in Table 2.

## 7. Conclusions

TNF, a key pro-inflammatory cytokine, plays a central role in the pathology of several types of dementia. Neuroinflammatory aspects of neurodegenerative diseases are related to various factors, both peripheral and located in the CNS. Pro-inflammatory cytokines prime glial cells, leading to aggravation of neuroinflammation. Unsurprisingly, lifestyle factors, such as diet, have a large impact on cognitive outcomes and should be considered as a crucial step in dementia prevention. So far, insufficient data exists on the use of TNF antagonists in dementia prevention and treatment. The predominance of studies conducted in AD models and the few experiments exploring their effects in other types of dementia may constitute a relevant research gap. Focused randomized clinical trials are warranted in order to establish the efficacy of anti-TNF agents and their mechanisms of action in most common types of dementia. It is still unknown, to what degree neuroinflammation resulting from COVID-19 may affect neurodegenerative processes and cognitive functioning in the long term with ongoing reports of chronic post-COVID complications. However, neuroinflammatory aspects of novel common infectious agents need to be taken into account in order to plan potential interventions focused on reduction in immune senescence in dementia treatment.

## Figures and Tables

**Table 1:** Summary of findings associated with diet and inflammation.

Study (Type)	Outcomes	Diet/Intervention	Group	Key Findings
Ostan et al., 2015 [126] (cohort study)	Inflammatory and metabolic parameters	RISTOMED diet (personalized and balanced) +/− nutraceutics	125 participants	RISTOMED diet alone or with each nutraceutical supplementation significantly decreased erythrocyte sedimentation rate
Kim et al., 2022 [127] (non-randomized intervention study)	Inflammatory parameters Insulin sensitivity	Short-term ketogenic diet (3 days)	15 participants	Short-term Ketogenic diet resulted in lower IL-1β and TNF secretion; Improved insulin sensitivity
Al-Abauidy et al., 2021 [128] (randomized clinical trial)	Oxidative stress and inflammatory parameters	Mediterranean diet (12 weeks)	19 participants	Mediterranean diet reduced IL-6 levels by 49% and levels of oxidative stress marker, 8-OHdG, by 32.4%
Georgoulis et al., 2021 [129] (randomized clinical trial)	Oxidative stress and inflammatory parameters	Mediterranean diet (6 months)	187 patients with obstructive sleep apnea	Mediterranean diet reduced hs-CRP levels in patients
Casas et al., 2017 [132] (randomized clinical trial)	Cytokine levels	Mediterranean diet +/− extra virgin olive oil (5 years)	66 participants	Mediterranean diet reduced IL-6, IL-8, MCP-1, and MIP-1β levels. Addition of extra virgin olive oil reduced IL-1β, IL-5, IL-7, IL-12p70, IL-18, TNF-α, IFN-γ, GCSF, GMCSF, and ENA78
Omorogieva et al., 2021 [130] (meta-analysis)	Lipid profiles, LPS, BMI, inflammatory markers	Diet rich in fiber	10 studies included in meta-analysis	Dietary fiber reduces total cholesterol, BMI and CRP, but no significant changes were observed for IL-6 and TNF
Shivappa et al., 2016 [131] (cross-sectional study)	Inflammatory markers	-	532 adolescents	Higher dietary inflammatory index scores were associated with increased levels of various inflammatory markers: TNF-α, IL-1, 2, IFN-γ and VCAM
Mazzoli et al., 2020 [133] (animal study)	Inflammatory markers, insulin sensitivity, BDNF	Western diet (4 weeks)	16 rats	Western diet increased TNF levels in white adipose tissue and hippocampus of rats; brain BDNF and synaptotagmin I were decreased, while PSD-95 was increased.
Jena et al., 2020 [134] (animal study)	Interleukin-17, PD-95, BDNF	High sugar and high fat diet (FPC diet) for 3 months, and 5 months +/− inulin supplementation	12 mice	FPC diet elevated RORγ and IL-17A signaling. Accompanied by microglia activation and reduced hippocampal long-term potentiation, FPC diet intake also reduced postsynaptic density-95 and brain derived neurotrophic factor.
Godfrey et al., 2020 [135] (animal study)	CRP levels, CSF dopamine concentrations Functional connectivity	12 months of obesogenic diet	34 female rhesus monkeys	CSF dopamine concentrations decreased, and CRP concentrations increased. Resting-state magnetic resonance neuroimaging showed that higher CRP concentrations were associated with decreased functional connectivity.

**Table 2:** Updates on clinical trials with the use of agents which potentially reduce neuroinflammation.

Anti-Inflammatory Agent	Clinicaltrial.gov Indentifier	Clinical Trial Phase	Results
Etanercept (TNF antagonist)	NCT01068353, NCT01716637, NCT00203359, NCT00203320	1–2	Etanercept was well tolerated and showed some trends toward cognitive, functional, and behavioral benefits
XPro1595/DN-TNF (TNF antagonist)	NCT03943264, NCT05321498, NCT05522387, NCT05318976	1,2	In phase 1 XPro1595 reduced white matter free water and increased the axonal integrity in adults with mild to moderate Alzheimer’s disease with signs of inflammation. Phase 2 trials are currently active
Dapagliflozin (selective sodium-glucose cotransporter 2 inhibitor)	NCT03801642	1/2	Trial ongoing; Alongside beneficial metabolic effects a potential anti-inflammatory effect via reduction in oxidative stress
ALZT-OP1/cromolyn + ibuprofen (mast cell stabilizer + NSAID)	NCT04570644, NCT02547818	1/2, 3	The combination of cromolyn and ibuprofen was safe and well tolerated. The concentrations of cromolyn and ibuprofen observed in the CSF are considered sufficient to titrate the estimated daily amyloid production and the associated inflammatory response in patients with AD. Phase 3 results are to be published.
Senicapoc (KCa3.1 blocker)	NCT04804241	2	Phase 2 trial is currently active. Previous animal studies show reduced neuroinflammation, decreased cerebral amyloid load, and enhanced hippocampal neuronal plasticity [164].
